# Physical Activity and Prevalence of Depression and Antidepressants in the Spanish Population

**DOI:** 10.3390/healthcare10020363

**Published:** 2022-02-12

**Authors:** Carmen Galán-Arroyo, Damián Pereira-Payo, Jorge Rojo-Ramos, Miguel A. Hernández-Mocholí, Eugenio Merellano-Navarro, Jorge Pérez-Gómez, Ángel Denche-Zamorano, Jose Carmelo Adsuar

**Affiliations:** 1 Promoting a Healthy Society Research Group (PHeSO), Faculty of Sport Sciences, University of Extremadura, 10003 Caceres, Spain; magaar04@alumnos.unex.es (C.G.-A.); jadssal@unex.es (J.C.A.); 2 Health Economy Motricity and Education (HEME), Faculty of Sport Science, University of Extremadura, 10003 Caceres, Spain; dpereirab@alumnos.unex.es (D.P.-P.); jorgepg100@unex.es (J.P.-G.); 3 Social Impact and Innovation in Health (InHEALTH), University of Extremadura, 10003 Caceres, Spain; jorgerr@unex.es; 4 Physical Activity and Quality of Life Research Group (AFYCAV), Faculty of Sport Science, University of Extremadura, 10003 Caceres, Spain; mhmocholi@unex.es; 5 Grupo de Investigación EFISAL, Universidad Autónoma de Chile, Talca 3460000, Chile; emerellano@gmail.com

**Keywords:** health, depression, physical activity, antidepressants, strength

## Abstract

Introduction: Depression is a mental disorder that affects more than 250 million people in the world, limiting their functional capacities. The work of public health policies is aimed at reducing its prevalence as well as its pharmaceutical cost. Physical activity (PA) programs are interventions with a high potential for effectiveness. Objectives: To establish the relationships between physical activity and the prevalence of depression and antidepressant intake in the Spanish population. Design: We performed a correlational study that was based on data from the European Health Survey Spain 2020 with 20,287 participants, aged 18–84 years, living in Spain. Results: Dependency relationships were found between the prevalence of depression, and: the frequency of PA, the number of days of PA per week, and the number of days of muscle strengthening in the population, in both sexes, and in all age groups (*p* < 0.001). Dependency relationships were found between the three PA variables and the prevalence of taking antidepressants (*p* < 0.001). An elevated prevalence of depression and antidepressant taking were found in the inactive groups compared to those who performed PA (*p* < 0.05). Conclusions: There is an inverse relationship between physical activity and the probability of suffering from depression and the intake of antidepressants. Performing PA 3–4 days/week, including 1–2 days of strength work, could be the best proposal to reduce the prevalence of depression in the Spanish population.

## 1. Introduction

Depression is currently the most prevalent mental disorder [1] and is characterized by profound sadness and loss of interest as well as a wide range of emotional, cognitive, physical and behavioral symptoms [2].

Depression is a major contributor to the global burden of disease [3], causing a substantial burden on health systems [4]. At its most extreme, it can lead to suicide [3]. Public health policies focus their efforts on preventing this disorder, which affects one in ten people in the world [1], 6.4% of Europeans [5] and 6.68% of Spanish [6]. There is a need to scale up strategies on early interventions [7], because treatments with antidepressants are not only costly but also unsuccessful [8]. This is done through health promotion campaigns, advice on good practices, etc. [1]. Health promotion through physical activity and exercise programs for adults is an intervention with a proven beneficial effect on reducing the risk of depression [1].

It is universally accepted that regular physical activity is one of the priorities in Public Health as a means of preventing chronic diseases [9] and the decrease in premature mortality [10]. By physical activity we mean any bodily movement that is produced by skeletal muscles that produces an energy expenditure that is greater than that at rest [11]. Regular physical activity improves both physical and mental health [12]. Closely linked to physical activity and Public Health is physical exercise, which is defined as planned, structured, and repeated physical activity that is aimed at acquiring, maintaining, or improving physical fitness [11]. In relation to PA and PE we can find strength work, which is physical ability to perform work or a movement, being one of the essential performance factors [13]. Muscle strengthening is closely related to health [14].

When it comes to depression, PA is an effective preventive tool against depressive disorders [15] and has a positive effect on physical and mental health due to the release of endorphins, reducing anxiety, depression, and stress [12].

There is a large body of evidence on the benefits of PA and PE for people with depression, in prevention [16,17] as a protective factor against depression [18], as a preventive strategy [19], effective in primary prevention [20], and treatment [21,22,23,24,25], and as an adjuvant treatment [19,26,27] from severe to moderate depression with an exercise program [28], where strengthening exercises [28] could reduce the intake of antidepressants [16,17]. There were two meta-analyses of structured exercise programs that were recently published that conclude that heart rate and muscle strength improvement (based on resistance exercise) are moderators of depression improvement in middle-aged and older adults [29,30]. However, there is a lack of documentation on the recommended frequency of physical activity depending on the type of exercise that is performed (aerobic and/or strength) to prevent or reduce the prevalence of depression and to establish strategies for different age groups.

The aim of this study is, therefore, to establish relationships between physical activity and the prevalence of depression and antidepressant intake in the Spanish population, and to be able to establish a recommended dose of physical exercise to prevent and reduce depression.

## 2. Materials and Methods

### 2.1. Study Design

In this cross-sectional investigation, a descriptive correlational study was performed that was based on data that were obtained from the public files included from the European Health Interview Survey of Spain 2020 (EESE 2020). The EESE is a survey that is conducted every five years by the Ministry of Health and the Spanish National Institute of Statistics (INE), forming part of the European Health Interview Survey (EHIS). This survey is coordinated by Eurostat and is regulated by Regulation (EC) 1338/2008 and Commission Regulation 141/2013. The interviews were conducted between 15 July 2019 to 24 July 2020 by previously trained and accredited interviewers.

### 2.2. Participants

The initial sample constituted of 22,072 participants, aged between 15 and 104, residing in Spain, who were interviewed on the occasion of the EESE 2020, individual adult questionnaire. For this study, data from people aged between 18 and 85 years were taken into account, so that those over 85 years of age (1282 participants) and those under 18 (503 participants) were excluded. The final sample was 20,287 persons: 9731 men and 10,556 women. 

The participants were selected using a three-stage sampling system with stratification: census sections (first stage units, municipalities were grouped into seven strata, according to number of inhabitants, selecting the sections in each stratum, taking into account the probability proportional to its size, according to the main family dwellings belonging to it), main family dwellings (second stage units, selecting living with equal probability, through systematic sampling with random start), and surveyable persons (third stage units, one person per dwelling, using the Kish random method. The selection is random, with equal probability for each adult in the dwelling). 

### 2.3. Ethics

Not required, since the data were obtained from anonymous, non-confidential public files.

### 2.4. Variables and Procedures

The variables that were used from EESE 2020 and the procedures that were followed with these variables are shown in the following table (Table 1).

### 2.5. Statistical Analysis

The data analysis was carried out with the IBM SPSS Statistics v.25 statistical analysis software.

Initially, the distribution that was followed by the data of the study variables was checked by performing a Kolgomorov–Smirnov test. When insufficient evidence was found to assume the normality of the variable distributions, it was accepted that the data did not follow a normal distribution for subsequent analyses. For this reason, in the descriptive analysis to characterize the sample, the median, the interquartile range (continuous variables), and the absolute and relative frequencies (ordinal variables) were used to present the data of the different groupings that were formed. Similarly, for this reason, nonparametric statistical tests were used to evaluate the possible intergroup differences using the Mann–Whitney U test (for the continuous variable: age). The chi-square statistic (to analyze possible dependency relationships), and a pairwise z-test for independent proportions, using the Bonferroni correction when it was necessary, (to analyze possible differences between proportions presented by sexes) were used for the categorical variables: age group, lifetime depression, 12-month depression, diagnosed depression, frequency of PA, days of PA, and days of muscle strengthening. The effect size was presented with Cramer’s V o phi as necessary.

The level of significance that was established in this investigation was less than 0.05.

## 3. Results

The median age of the population that was studied was 54 years, being lower in men (53) than in women (55), with significant differences between the two (*p* < 0.001). Similarly, dependency relationships were found between the age group and sex (*x*^2^ = 57.97, *p* < 0.001, *V* = 0.053), with women presenting a higher proportion of the population than men in the elderly group (Table 2).

The prevalence of depression that was suffered at some time in life was 9.5% in the general population. Dependence relationships were found between the prevalence of depression and sex (*x*^2^ = 234.80, *p* < 0.001, *Φ* = 0.108), with women (12.5%) having a higher prevalence than men (6.2%), with significant differences between them (*p* < 0.05). Similar results were found in the prevalence of depression in the last 12 months (7.0%) and diagnosed by a physician (8.6%) in the general population, with dependence relationships between the prevalence and the sex of the participants (*x*^2^ = 187.90, *p* < 0.001, *Φ* = 0.096 and *x*^2^ = 234.28, *p* < 0.001, *Φ* = 0.108, respectively). In both, significant differences were found between the sexes (*p* < 0.05), with 6 and 4.6 percentage points of difference between the men and the women, with higher prevalence in women, both in depression at 12 months, as well as in diagnosed depression. These dependency relationships were also found between the prevalence of antidepressant use and sex (*x*^2^ = 206.74, *p* < 0.001, *Φ* = 0.101). The prevalence of taking antidepressants in the general population was 5.5%, being more than twice as high in women (7.7%) than in men (3.1%), with statistically significant differences in prevalence (*p* < 0.05) (Table 2). 

A total of 74.6% of the general population reported not doing PA on a regular frequency: never (35.4%) and occasionally (39.2%). Dependency relationships were found between the frequency of PA and sex (*x*^2^ = 144.17, *p* < 0.001, *V* = 0.084), the proportion of inactive women (38.3%) was 5.2 percentage points higher than that of men (33.1%), and the difference between the proportions of people with a frequency of several times a month or more between men (28.8%) and women (22.2%) was 6.6 points; all of these differences were significant (*p* < 0.05). Dependency relationships were also found between the days of PA per week and sex (*x*^2^ = 82.96, *p* < 0.001, *V* = 0.064). A total of 51.6% of the population reported performing PA zero days a week, this proportion being higher in women (54.7%) than in men (48.8%), with significant differences between the two (*p* < 0.05). Something that was also found in the proportions of people with 5+ days of PA, being 18.9% in the general population, and finding a difference of 4 percentage points between men (21%) and women (17%). Much higher were the proportions of people who reported not performing muscle-strengthening exercises on any day of the week. In this sense, 81.8% of the general population performed these activities on zero days, and there was a relationship of dependence between these activities and sex (*x*^2^ = 118.97, *p* < 0.001, *V* = 0.077). In women, this prevalence reached 83.9% compared to 79.6% in men, with *p* < 0.05 (Table 2).

Dependency relationships were found between the lifetime prevalence of depression and the frequency of PA, both in the general population (*x*^2^ = 235.15, *p* < 0.001, *V* = 0.108) and in both sexes (Men: *x*^2^ = 76.73, *p* < 0.001, *V* = 0.089; Women: *x*^2^ = 126.85, *p* < 0.001, *V* = 0.110). The prevalence of lifetime depression was 13.4% in the inactive general population, being 4.8 percentage points lower in the population that performed PA occasionally (8.6%) and about 8 points lower in the more active groups (5.2% in the group that performed PA several times a month and 5.5% in the group that performed PA several times a week), finding significant differences between the groups (*p* < 0.05). The same occurred in men and women, with significant differences between the prevalence of inactive and occasional people, and between these and the higher levels (*p* < 0.05). In women, the highest prevalence of lifetime depression was found in inactive women (16.7%), with women who performed PA several times a month having the lowest prevalence (7.1%). This was similar for men, with the lowest prevalence found among men who performed PA several times a month (3.6%). These dependency relationships, both in the general population and in both sexes, were found in the prevalence of depression in the last 12 months and of diagnosed depression. Thus, significant differences were also found between the prevalence of depression among the different PA groups, being higher in the inactive than in the occasional, and in both groups and higher PA levels, with *p* < 0.05. On the other hand, the prevalence of taking antidepressants showed dependence relationships with the frequency of PA, both in the general population (*x*^2^ = 198.95, *p* < 0.001, *V* = 0.099) and in both sexes (Men: *x*^2^ = 72.52, *p* < 0.001, *V* = 0.086; Women: *x*^2^ = 102.33, *p* < 0.001, *V* = 0.099). The prevalence in the inactive population was more than three times higher than the prevalence in the group with the highest frequency of PA (8.2% vs. 2.5), with significant differences in proportions between these groups (*p* < 0.05). This finding in the general population also occurred in both men and women. Men who performed PA several times a month had the lowest prevalence of taking antidepressants (1.3%), while the highest prevalence was found in inactive men (5.1%). On the other hand, inactive women had a prevalence that was 6.9 points higher than women with the highest frequency of PA (10.7% vs. 3.8) (Table 3). 

The prevalence of lifetime, past 12 months, and physician-diagnosed depression also presented dependence relationships with the days of PA that were performed per week, both in the general population and in both sexes. The highest prevalence in the three depression conditions were in the groups that performed zero days of PA per week compared to the groups that performed PA, at least one day, with *p* < 0.05; this was found in the general population, in men and in women. The lowest prevalence was found in the groups that performed PA between three and four days a week, with significant differences, in most cases, with respect to the prevalence of the other groups (*p* < 0.05). The highest prevalence was found in the inactive women: lifetime depression (15.7%), 12-month depression (12.1%), and diagnosed depression (14.5%); with differences in proportions between 6–7 points with respect to the women who performed PA three to four days a week (lifetime: 7.8%; 12-months: 5.3%; diagnosed: 7.1%) who presented the lowest prevalence. With smaller differences in proportions, the same was found in men, who presented differences of around 4–5 points between inactive and men with PA three to four days a week. These dependency relationships and these differences between the PA groups were also found in the prevalence of taking antidepressants, with prevalence that doubled or even tripled between inactive persons and those who took PA three to four days a week (Table 4).

The existence of dependency relationships between the prevalence of the three conditions of depression that were analyzed in this study and the number of days per week that muscle-strengthening activities were also found, both in the general population and in both sexes. In men, significant differences were found between the prevalence of depression of those who did not perform, at least one day a week, muscle strengthening exercises, compared to the rest of the groups, although no differences were found between the rest of the groups among themselves (*p* < 0.05). In women, the lowest prevalence of depression in the three conditions was found in those who performed these activities one to two days a week. Finally, dependency relationships were also found between the prevalence of taking antidepressants and the days per week that muscle strengthening exercises were performed, finding significant differences between those who did not perform, at least one day, and the rest (*p* < 0.05) in the general population (Table 5).

In each age group, the prevalence of depression that was suffered at any time in life was found to be related to the frequency of PA. In the young general population, the prevalence of depression reached 3.2%. In inactive young people, the prevalence was found to be 2.1 percentage points higher than the average (5.3%), while the prevalence decreased to 1.3% in those who performed PA several times a month. In the elderly, the prevalence in inactive persons was 19.8%, 10.6 percentage points higher than in the elderly with PA several times a month (9.2%). In any age group, significant differences were found in the prevalence of depression at any time of life between inactive persons and the rest of the PA groups, although not between them (*p* < 0.05). The same relationships and differences were found in the prevalence of diagnosed depression. On the other hand, although the previous dependency relationships were again found in the prevalence of depression in the last 12 months in all age groups, in adults and the elderly, differences were found between performing PA occasionally and performing PA several times a month, or more, with lower prevalence in the latter (*p* < 0.05). In the elderly, the prevalence of depression in the last 12 months was tripled in inactive persons (15.7%) compared to those who performed PA several times a month (4.7%), with *p* < 0.05. Regarding the prevalence of taking antidepressants, dependence relationships were found with the frequency of PA in all age groups. In adults and the elderly, significant differences were found between the prevalence of inactive people and the rest of the groups (*p* < 0.05) (Table 6).

The results were not very different when analyzing the relationships between the prevalence of depression in each of the conditions and the PA groups, according to the days per week. Dependency relationships were found in all of them and in all age groups. A lower prevalence was found in all groups with one PA day per week, or more, compared to the inactive group, with *p* < 0.05. In young people, between the inactive (5.3%) and the groups with the highest number of PA days per week reached 3.6 percentage points, these differences reached 8 percentage points in the elderly (0 days/week: 17.2% vs. 5+days/week: 9.2%) in the prevalence of depression that was suffered throughout life, with similar differences in the prevalence of depression in the last 12 months and in diagnosed depression. The prevalence of taking antidepressants was also related to the days of PA per week in all age groups. Although significant differences were found between then inactive and the rest of the groups, no differences were found in the latter among themselves (*p* < 0.05). However, in young people and the elderly, no differences were found between the inactive people and those who performed PA 1–2 days a week, or between these and the rest of the groups (*p* < 0.05) (Table 7).

Finally, dependency relationships were found between the prevalence of depression in the three conditions that were analyzed and the days per week of muscle strengthening activities in all the age groups. In all the age groups, significant differences were found between the prevalence of the inactive groups and those who performed strengthening activities one to two times per week (*p* < 0.05), being lower in the latter. However, the same did not occur with the prevalence of all the groups with a greater number of days per week, finding increased prevalence with respect to those of the one to two days per week group. Similarly, dependence relationships were found between the prevalence of taking antidepressants and the number of days of strengthening activities in all the age groups, although significant differences were only found between some groups, with the highest prevalence in the group of young people in the group of five days or more per week, although without significant differences with the inactive group. In the rest of the age groups, although with lower prevalence, the differences between the two groups were not significant either, although they were significant with other groups (Table 8).

## 4. Discussion

The first finding of our study was in relation to sex. Significant differences were found (*p* < 0.05), as noted in most studies, where being female is a risk factor that is associated with depression [31,32]. Likewise, dependency relationships were found between age group and sex, with women presenting a higher proportion of the population with depression than men in the elderly group; this is consistent with the majority of studies indicating that depression is higher in older women [33,34,35].

Regarding age, the median age of the population that was studied was 54 years, being lower in men (53) than in women (55), with significant differences between the two (*p* < 0.001). It may be related to the onset of menopause in women, as indicated by other studies [36,37].

The prevalence of depression that was suffered at some point in life was 9.5% in the general population, the same as the ENSE 2017 survey, whose figure stands at 9.2%. Dependent relationships were found between the prevalence of depression and sex, with women (12.5%) presenting a prevalence twice that of men (6.2%), with significant differences between the two (*p* < 0.05). Similar results were found in the prevalence of depression in the last 12 months and diagnosed by a physician (8.6%) in the general population, as noted in most studies, where being female is a risk factor that is associated with depression [31,32].

Also, dependence relationships were found between the prevalence of taking antidepressants and sex, being more than twice as high in women (7.7%) than in men (3.1%), with statistically significant differences between both (*p* < 0.05). This is in line with other studies [38,39] where the prevalence of antidepressant use is twice as high in women as in men.

Similarly, dependency relationships were found between PA frequency and sex. The rate of inactive women is higher than that of men, as reported in other studies [40,41]. In terms of strength work, women perform less strengthening exercises with *p* < 0.05 [42].

Another important finding is that dependence relationships were found between the prevalence of depression (lifetime, in the last 12 months and diagnosed) and the frequency of PA, both in the general population and in both sexes. The higher the frequency of physical activity, the lower the prevalence of depression. There are several studies and systematic reviews supporting this finding [43,44,45]. The prevalence of taking antidepressants showed dependence relationships with the frequency of PA; in this line there is another study where the probability of taking antidepressants increases with inactivity [46].

The existence of dependency relationships between the prevalence of the three depression conditions that were analyzed in this research and the number of days per week that muscle strengthening activities were performed were also found, both in the general population and in both sexes; coinciding with the systematic review on physical exercise and depression [47,48]. In all the age groups, significant differences were found between the prevalence of the inactive groups and those who performed strengthening activities one to two times per week (*p* < 0.05), being lower in the latter. However, the same did not occur with a higher number of days per week, with increased prevalence.

If we know the recommended frequency of physical activity and the type of exercise to prevent or reduce the prevalence of depression in different age groups, our findings could be a reference for monitoring the prevalence of current depressive disorders, planning health resources and services, and developing screening and preventive strategies in different age groups at the national level.

### 4.1. Theoretical and Practical Implications

Health education and promotion campaigns among the Spanish population could help to reduce the prevalence of depression in all the age groups. Increasing the frequency of PA in inactive people or those with low levels of PA to three to four days/week of moderate physical activity, including one to two days of strength work, could reduce the prevalence of depression and antidepressant use in the Spanish population.

Possible initiatives that governmental policies could carry out could be: from including active breaks in high schools and universities for the youth group; physical activity programs in companies for adults, to active aging programs for the elderly.

### 4.2. Limitations

This article has some limitations to take into account: it is cross-sectional in nature. The data were obtained through the information that was submitted by the participants. Cause-effect relationships cannot be established due to the methodology that was used. Only male and female sex is considered; non-binary sex is not taken into account. Physical activity measures were not objectively assessed. Only whether or not participants were taking antidepressants was recorded; neither the active ingredient nor the amount taken by each participant who reported taking antidepressants was recorded. Other variables that could affect depression, such as sociodemographic, socioeconomic, and the sociocultural biases of the participants, were also not included, because although there are studies in which these variables seem to affect depression [49,50], this greatly reduced the sample by including new divisions in the groups, losing statistical power.

## 5. Conclusions

There is an inverse relationship between physical activity and the probability of suffering from depression and the intake of antidepressants. The higher the frequency of physical exercise, the lower the prevalence of depression and the lower the intake of antidepressants. Performing PA three to four days/week, including one to two days of strength work, could be the best proposal to reduce the prevalence of depression in the Spanish population.

## Figures and Tables

**Table 1:** Variables and definitions.

Variable	Definition of Variable
Gender	Male or female.
Age	In years.
Age group	Youth (18–34 years); Young adults (35–49 years); Older adults (50–64 years); Older (65–84 years).
Life Depression	Item p.25_20a: “Have you ever suffered from depression?” Yes (Depression). No (No depression). For the analyses that included this variable, 19 participants with responses, Don’t know, or No answer (NS/NC) were excluded.
Depression 12 months	Item p.25_20b: “Have you suffered from it in the last 12 months?” Yes (Depression). No (No depression). For analyses including this variable, 22 participants with responses, NS/NC.
Diagnosed Depression	Item p.25_20c: “Has a doctor told you that you have it?” Yes (Depression). No (No depression). For analyses that included this variable, 21 participants with “NS/NC” responses were excluded.
Antidepressants	From items: Q.85: “During the past 2 weeks, have you taken any medication that was prescribed to you by a physician?” Yes/No; Q86: “During the past 2 weeks, have you taken any medications, including herbal medications or vitamins, that were not prescribed to you by a doctor?” Yes/No; Q87_14a: “Next, I am going to read you a list of types of medications, please tell me which one(s) of them you have taken in the last 2 weeks—antidepressants, stimulants?” Yes/No. Antidepressants (Participants with affirmative answers to items p.87_14a); Non-depressants (participants with “No” in items: p.85 and p.86; or with these, “Yes”, if p.87_14a = “No”).
Frequency of PA	From item p.112: “Which of these possibilities best describes the frequency with which you do some physical activity in your free time?” Never (“*I do not exercise. I spend my free time almost completely sedentary”*); Occasional (“*I do some occasional physical or sport activity”*), Several a month *(“I do physical activity several times a month”*); Several a week (*“I do sport or physical training several times a week”*).
PA days	From item p.117: “How many days do you practice sport, gymnastics, cycling, fast walking, etc., at least 10 min in a row?”. Groups were created: 0 days/week, 1–2 days/week, 3–4 days/week and 5+ days/week.
Muscle strengthening days	From item p.119: “How many days do you do activities specifically aimed at strengthening your muscles?” Groups were created: 0 days/week, 1–2 days/week, 3–4 days/week and 5+ days/week.

**Table 2:** Sociodemographic characteristics: age, age group, prevalence of depression and of taking antidepressants and physical activity; of the Spanish population aged 18–84 years in the EESE 2020.

Variables							
Ages (Years)	Total = 20,287	Men *n* = 9731	Women *n* = 10,556	-	-	*p*	-
Median (RI)	54 (26)	53 (24)	55 (27)	-	-	<0.001	-
Median (DE)	53.4 (16.8)	52.7 (16.5)	54.1 (17.0)	-	-	-	-
Age (Years)	Total = 20,287	Men *n* = 9731	Women *n* = 10,556	*x* ^2^	*df*	p*	*V*
Young people	2913 (14.4)	1409 (14.5)	1504 (14.2)	58.0	4	<0.001	0.053
Young adults	5647 (27.8)	2841 (29.2)	2806 (26.6)				
Older adults	5842 (28.8)	2881 (29.6)	2961 (28.1)				
Older	5885 (29.5)	2600 (26.7) a	3285 (31.1) b				
Depression Life	Total = 20,268	Men *n* = 9726	Women *n* = 10,542	*x* ^2^	*df*	p*	*Φ*
Depression	1922 (9.5)	603 (6.2) a	1319 (12.5) b	234.8	1	<0.001	0.108
No depression	18,346 (90.5)	9123 (93.8) a	9223 (87.5) b				
Depression 12 months	Total = 20,265	Me n*n* = 9725	Women *n* = 10,540	*x* ^2^	*df*	p*	*Φ*
Depression	1411 (7.0)	429 (4.4) a	982 (9.3) b	187.90	1	<0.001	0.096
No depression	18,854 (93.0)	9296 (95.6) a	9558 (90.7) b				
Diagnosed Depression	Total = 20,266	Men *n* = 9725	Women *n* = 10,541	*x* ^2^	*df*	p*	*Φ*
Depression	1750 (8.6)	534 (5.5) a	1216 (11.5) b	234.28	1	<0.001	0.108
No depression	18,516 (91.4)	9191 (94.5) b	9325 (88.5) b				
Antidepressants	Total = 20,280	Men *n* = 9727	Women *n* = 10,553	*x* ^2^	*df*	p*	*Φ*
Antidepressants	1116 (5.5)	302 (3.1) a	814 (7.7) b	206.74	1	<0.001	0.101
No antidepressants	19,164 (94.5)	9425 (96.9) a	9739 (92.3) b				
Frequency of PA	Total = 20,269	Men *n* = 9722	Women *n* = 10,547	*x* ^2^	*df*	p*	*V*
Never	7168 (35.4)	3124 (32.1) a	4044 (38.3) b	144.17	3	<0.001	0.084
Occasionally	7955 (39.2)	3793 (39.0) a	4162 (39.5) a				
Several per month	2134 (10.5)	1151 (11.8) a	983 (9.3) b				
Several per week	3012 (14.9)	1654 (17.0) a	1358 (12.9) b				
Days of PA	Total = 20,163	Men *n* = 9673	Women *n* = 10,490	*x* ^2^	*df*	p*	*V*
0 days/week	10,412 (51.6)	4725 (48.8) a	5687 (54.2) b	82.96	3	<0.001	0.064
1–2 days/week	2508 (12.4)	1175 (12.1) a	1333 (12.7) a				
3–4 days/week	3426 (17.0)	1740 (18.0) a	1686 (16.1) b				
5+ days/week	3817 (18.9)	2033 (21.0) a	1784 (17.0) b				
Strengthening days	Total = 20,104	Men *n* = 9642	Women *n* = 10,462	*x* ^2^	*df*	p*	*V*
0 days/week	16,452 (81.8)	7673 (79.6) a	8779 (83.9) b	118.97	3	<0.001	0.077
1–2 days/week	1467 (7.3)	681 (7.1) a	786 (7.5) a				
3–4 days/week	1391 (6.9)	813 (8.4) a	578 (5.5) b				
5+ days/week	794 (3.9)	475 (4.9) a	319 (3.0) b				

*p* (*p*-value. Mann–Whitney U); p* (*p*-value. Chi square statistic); *df* (degree freedom); *Φ* (phi); *V* (Cramer’s V); RI (Interquartile range); SD (Standard deviation); The data are presented by absolute and relative frequencies (ordinal variables); Depression (Depression diagnosed by a physician); Non-depression (Depression no diagnosed); Antidepressants (have taken antidepressants in the last two weeks); No antidepressants (have not taken antidepressants in the last two weeks); Frequency of PA (“Which of these possibilities best describes the frequency with which you do some physical activity in your free time?”); Days PA (how many days do you practice sport, gymnastics, cycling, brisk walking, etc., at least 10 min at a time); Strengthening days (how many days do you do activities specifically aimed at strengthening your muscles); ab (Each subscript corresponds to significant differences between column proportions at 95%).

**Table 3:** Relationship between the prevalence of: depression (lifetime, in the last 12 years and diagnosed); consumption of antidepressants; and frequency of physical activity (T112) in the general Spanish population of the EESE 2020 between 18 and 84 years, and by sex.

Frequency of Physical Activity										
Sex	Depression throughout Life	Total	Never	Occasional	Several/Month	Several/Week	*x* ^2^	*df*	p*	*V*
Men (*n* = 9717)	Depression	603 (6.2)	284 (9.1) a	215 (5.7) b	41 (3.6) c	63 (3.8) c	76.73	3	<0.001	0.089
	No depression	9114 (93.8)	2828 (90.9) a	3578 (94.3) b	1108 (96.4) c	1590 (96.2) c				
Women (*n* = 10,535)	Depression	1315 (12.5)	674 (16.7) a	469 (11.3) b	70 (7.1) c	102 (7.5) c	126.85	3	<0.001	0.110
	No depression	9220 (87.5)	3367 (83.3) a	3688 (88.7) b	912 (92.9) c	1253 (13.6) c				
Total (*n* = 20,252)	Depression	1918 (9.5)	958 (13.4) a	684 (8.6) b	111 (5.2) c	165 (5.5) c	235.15	3	<0.001	0.108
	No depression	18,334 (90.5)	6205 (86.6) a	7266 (91.4) b	2020 (94.8) c	2843 (94.5) c				
Sex	Depression last 12 months	Total	Never	Occasional	Several/month	Several/week	*x* ^2^	*df*	p*	*V*
Men (*n* = 9716)	Depression	429 (4.4)	221 (7.1) a	150 (4.0) b	22 (1.9) c	36 (2.2) c	91.00	3	<0.001	0.097
	No depression	9287 (95.6)	2901 (92.9) b	3642 (96.0) b	1127 (98.1) c	1617 (97.8) c				
Women (*n* = 10,533)	Depression	978 (9.3)	523 (12.9) a	344 (8.3) b	42 (4.3) c	69 (5.1) c	126.80	3	<0.001	0.110
	No depression	9555 (90.7)	3517 (87.1) a	3812 (91.7) b	940 (95.7) c	1286 (94.9) c				
Total (*n* = 20,249)	Depression	1407 (6.9)	744 (10.4) a	494 (6.2) b	64 (3.0) c	105 (3.5) c	244.58	3	<0.001	0.110
	No depression	18,842 (93.1)	6418 (89.6) a	7454 (93.8) b	2067 (97.0) c	2903 (96.5) c				
Sex	Diagnosed depression	Total	Never	Occasional	Several/month	Several/week	*x* ^2^	*df*	p*	*V*
Men (*n* = 9716)	Depression	534 (5.5)	254 (8.1) a	187 (4.9) b	35 (3.0) c	58 (3.5) c	70.14	3	<0.001	0.085
	No depression	9182 (94.5)	2867 (91.9) a	3606 (95.1) b	1114 (97.0) c	1595 (96.5) c				
Women (*n* = 10,534)	Depression	1213 (11.5)	631 (15.6) a	424 (10.2) b	65 (6.6) c	93 (6.9) c	125.71	3	<0.001	0.109
	No depression	9321 (88.5)	3409 (84.4) a	3733 (89.8) b	917 (93.4) c	1262 (93.1) c				
Total (*n* = 20,250)	Depression	1747 (8.6)	885 (12.4) a	611 (7.7) b	100 (4.7) c	151 (5.0) c	226.93	3	<0.001	0.106
	No depression	18,503 (91.4)	6276 (87.6) a	7339 (92.3) b	2031 (95.3) c	2857 (95.0) c				
Sex	Antidepressants	Total	Never	Occasional	Several/month	Several/week	*x* ^2^	*df*	p*	*V*
Men (*n* = 9718)	Antidepressants	302 (3.1)	160 (5.1) a	104 (2.7) b	17 (1.5) c	21 (1.3) c	72.52	3	<0.001	0.086
	No antidepressants	9416 (96.9)	2963 (94.9) a	3686 (97.3) b	1134 (98.5) c	1633 (98.7) c				
Women (*n* = 10,545)	Antidepressants	813 (7.7)	431 (10.7) a	293 (7.0) b	37 (3.8) c	52 (3.8) c	102.33	3	<0.001	0.099
	No antidepressants	9732 (92.3)	3612 (89.3) a	3868 (93.0) b	946 (96.2) c	1306 (96.2) c				
Total (*n* = 20,263)	Antidepressants	1115 (5.5)	591 (8.2) a	397 (5.0) b	54 (2.5) c	73 (2.4) c	198.95	3	<0.001	0.099
	No antidepressants	19,148 (94.5)	6575 (91.8) a	7554 (95.0) b	2080 (97.5) c	2939 (97.6) c				

The data presented by absolute and relative frequencies; Depression (Depression diagnosed by a physician); Non-depression (Depression no diagnosed); Antidepressants (have taken antidepressants in the last two weeks); No antidepressants (have not taken antidepressants in the last two weeks); Frequency of PA (“Which of these possibilities best describes the frequency with which you do some physical activity in your free time?”); p* (*p*-value. Chi-square statistic); *df* (degree freedom); (Cramer’s V); abc (Each subscript corresponds to significant differences between column proportions at 95%); *n* (participants).

**Table 4:** Relationship between the prevalence of depression (lifetime, in the last 12 years and diagnosed) and the number of days of physical activity per week in the general Spanish population of the EESE 2020 between 18 and 84 years of age, and by sex.

Days of Physical Activity per Week										
Sex	Depression Life	Total	0 days/week	1–2 days/week	3–4 days/week	5+ days/week	*x* ^2^	*df*	p*	*V*
Men (*n* = 9668)	Yes	600 (6.2)	391 (8.3) a	47 (4.0) b.c	57 (3.3) c	105 (5.2) b	74.07	3	<0.001	0.088
	No	9068 (93.8)	4332 (91.7) a	1128 (96.0) b.c	1683 (96.7) c	1925 (94.8) b				
Women (*n* = 10,478)	Yes	1312 (12.5)	891 (15.7) a	134 (10.1) b	131 (7.8) c	156 (8.8) b.c	116.83	3	<0.001	0.106
	No	9166 (ç87.5)	4790 (84.3) a	1198 (89.9) b	1553 (92.2) c	1625 (91.2) b.c				
Total (*n* = 20,252)	Yes	1912 (9.5)	1282 (12.3) a	181 (7.2) b	188 (5.5) c	261 (6.8) b	206.90	3	<0.001	0.101
	No	18,234 (90.5)	9122 (87.7) a	2326 (92.8) b	3236 (94.5) c	3550 (93.2) b				
Sex	Depression 12 months	Total	0 days/week	1–2 days/week	3–4 days/week	5+ days/week	*x* ^2^	*df*	p*	*V*
Men (*n* = 9677)	Yes	426 (4.4)	297 (6.3) a	30 (2.6) b.c	36 (2.1) c	63 (3.1) b	80.02	3	<0.001	0.091
	No	9241 (95.6)	4426 (93.7) a	1145 (97.4) b.c	1704 (97.9) c	1966 (96.9) b				
Women (*n* = 10,476)	Yes	976 (9.3)	688 (12.1) a	89 (6.7) b	90 (5.3) b	109 (6.1) b	116.44	3	<0.001	0.105
	No	9500 (90.7)	4992 (87.9) a	1243 (93.3) b	1594 (94.7) b	1671 (93.9) b				
Total (*n* = 20,143)	Yes	1402 (7.0)	985 (9.5) a	119 (4.7) b	126 (3.7) c	172 (4.5) b.c	212.08	3	<0.001	0.103
	No	18,741 (93.0)	9418 (90.5) a	2388 (95.3) b	3298 (96.3) c	3637 (95.5) b.c				
Sex	Diagnosed Depression	Total	0 days/week	1–2 days/week	3–4 days/week	5+ days/week	*x* ^2^	*df*	p*	*V*
Men (*n* = 9667)	Yes	532 (5.5)	349 (7.4) a	38 (3.2) b.c	51 (2.9) c	94 (4.6) b	69.10	3	<0.001	0.085
	No	9135 (94.5)	4373 (92.6) a	1137 (96.8) b.c	1689 (97.1) c	1936 (95.4) b				
Women (*n* = 10,477)	Yes	1209 (11.5)	825 (14.5) a	127 (9.5) b	120 (7.1) c	137 (7.7) b.c	112.79	3	<0.001	0.104
	No	9268 (88.5)	4855 (85.5) a	1205 (90.5) b	1564 (92.9) c	1644 (92.3) b.c				
Total (*n* = 20,144)	Yes	1741 (8.6)	1174 (11.3) a	165 (6.6) b	171 (5.0) c	231 (6.1) b	195.44	3	<0.001	0.099
	No	18,403 (91.4)	9228 (88.7) a	2342 (93.4) b	3253 (95.0) c	3580 (93.9) b				
Sex	Antidepressants	Total	0 days/week	1–2 days/week	3–4 days/week	5+ days/week	*x* ^2^	*df*	p*	*V*
Men (*n* = 9669)	Yes	301 (3.1)	215 (4.6) a	16 (1.4) b	25 (1.4) b	45 (2.2) b	66.12	3	<0.001	0.083
	No	9368 (96.9)	4506 (95.4) a	1159 (98.6) b	1715 (98.6) b	1988 (97.8) b				
Women (*n* = 10,488)	Yes	810 (7.7)	560 (9.9) a	88 (6.6) b	77 (4.6) c	85 (4.8) c	83.93	3	<0.001	0.089
	No	9678 (92.3)	5125 (90.1)	1245 (93.4) b	1609 (95.4) c	1699 (95.2) c				
Total (*n* = 20,157)	Yes	1111 (5.5)	775 (7.4) a	104 (4.1) b	102 (3.0) c	130 (3.4) b.c	158.62	3	<0.001	0.089
	No	19,046 (94.5)	9631 (92.6) a	2404 (95.9) b	3324 (97.0) c	3687 (96.6) b.c				

The data are presented by absolute and relative frequencies; Depression (Depression diagnosed by a physician); Non-depression (Depression no diagnosed); Antidepressants (have taken antidepressants in the last 2 weeks); No antidepressants (have not taken antidepressants in the last two weeks); Days PA (how many days do you practice sport, gymnastics, cycling, brisk walking, etc., at least 10 min at a time); p* (*p*-value. Chi-square statistic); *df* (degree freedom); *V* (Cramer’s V); abc (Each subscript corresponds to significant differences between column proportions at 95%); *n* (participants).

**Table 5:** Relationship between the prevalence of depression (lifetime, in the last 12 years and diagnosed) and days of muscle-strengthening training per week in the general Spanish population of EESE 2020 between 18 and 84 years of age, and by sex.

Muscle Strengthening Days per Week										
Sex	Depression Life	Total	0 days/week	1–2 days/week	3–4 days/week	5+ days/week	*x* ^2^	*df*	p*	*V*
Men (*n* = 9637)	Yes	600 (6.2)	527 (6.9) a	22 (3.2) b	31 (3.8) b	20 (4.2) b	27.27	3	<0.001	0.053
	No	9037 (93.8)	7143 (93.1) a	659 (96.8) b	781 (96.2) b	454 (95.8) b				
Women (*n* = 10,450)	Yes	1311 (12.5)	1210 (13.8) a	39 (5.0) b	41 (7.1) b	21 (6.6) b	79.53	3	<0.001	0.087
	No	9139 (87.5)	7559 (86.2) a	746 (95.0) b	536 (92.9) b	298 (93.4) b				
Total (*n* = 20,087)	Yes	1911 (9.5)	1737 (10.6) a	61 (4.2) b	72 (5.2) b	41 (5.2) b	117.58	3	<0.001	0.077
	No	18,176 (90.5)	14,702 (89.4) a	1405 (7.7) b	1317 (94.8) b	752 (94.8) b				
Sex	Depression 12 months	Total	0 days/week	1–2 days/week	3–4 days/week	5+ days/week	*x* ^2^	*df*	p*	*V*
Men (*n* = 9636)	Yes	426 (4.4)	383 (5.0) a	14 (2.1) b	18 (2.2) b	11 (2.3) b	29.22	3	<0.001	0.055
	No	9210 (95.6)	7287 (95.0) a	667 (97.9) b	794 (97.8) b	462 (97.7) b				
Women (*n* = 10,448)	Yes	976 (9.3)	906 (10.3) a	21 (2.7) b	34 (5.9) c	15 (4.7) b.c	67.51	3	<0.001	0.080
	No	9472 (90.7)	7862 (89.7) a	764 (97.3) b	543 (94.1) c	303 (95.3) b.c				
Total (*n* = 20,084)	Yes	1402 (7.0)	1289 (7.8) a	35 (2.4) b	52 (3.7) c	26 (3.3) b.c	105.43	3	<0.001	0.072
	No	18,682 (93.0)	15,149 (92.2) a	1431 (97.6) b	1337 (96.3) c	765 (96.7) b.c				
Sex	Diagnosed Depression	Total	0 days/week	1–2 days/week	3–4 days/week	5+ days/week	*x* ^2^	*df*	p*	*V*
Men (*n* = 9636)	Yes	532 (5.5)	463 (6.0) a	18 (2.6) b	31 (3.8) b	20 (4.2) a.b	20.79	3	<0.001	0.046
	No	9104 (94.5)	7206 (94.0) a	663 (97.4) b	781 (96.2) b	454 (95.8) a.b				
Women (*n* = 10,449)	Yes	1208 (11.6)	1121 (12.8) a	33 (4.2) b	39 (6.8) c	15 (4.7) b.c	82.10	3	<0.001	0.089
	No	9241 (88.4)	7647 (87.2) a	752 (95.8) b	538 (93.2) c	304 (95.3) b.c				
Total (*n* = 20,085)	Yes	1740 (8.7)	1584 (9.6) a	51 (3.5) b	70 (5.0) c	35 (4.4) b.c	110.62	3	<0.001	0.074
	No	18,345 (91.3)	14,853 (90.4) a	1415 (96.5) b	1319 (95.0) c	758 (95.6) b.c				
Sex	Antidepressants	Total	0 days/week	1–2 days/week	3–4 days/week	5+ days/week	*x* ^2^	*df*	p*	*V*
Men (*n* = 9638)	Yes	301 (3.1)	268 (3.5) a	13 (1.9) b	11 (1.4) b	9 (1.9) a.b	17.60	3	<0.001	0.043
	No	9368 (96.9)	7401 (96.5) a	668 (98.1) b	802 (98.6) b	466 (98.1) a.b				
Women (*n* = 10,460)	Yes	809 (7.7)	743 (8.5) a	25 (3.2) b	23 (4.0) b	18 (5.6) a.b	42.79	3	<0.001	0.064
	No	9651 (92.3)	8034 (91.5) a	761 (96.8) b	555 (96.0) b	301 (94.4) a.b				
Total (*n* = 20,098)	Yes	1111 (5.5)	1011 (6.1) a	38 (2.6) b	34 (2.4) b	27 (3.4) b	68.59	3	<0.001	0.058
	No	19,046 (94.5)	15,435 (93.8) a	1429 (97.4) b	1357 (97.6) b	767 (96.6) b				

The data are presented by absolute and relative frequencies; Depression (Depression diagnosed by a physician); Non-depression (Depression no diagnosed); Antidepressants (have taken antidepressants in the last two weeks); No antidepressants (have not taken antidepressants in the last two weeks); Strengthening days (how many days do you do activities specifically aimed at strengthening your muscles); p* (*p*-value. Chi-square statistic); *df* (degree freedom); *V* (Cramer’s V); abc (Each subscript corresponds to significant differences between column proportions at 95%); *n* (participants).

**Table 6:** Relationship between the prevalence of depression (lifetime, past 12 years and diagnosed) and the frequency of physical activity in the general Spanish population of EESE 2020 between 18 and 84 years, by age group.

Frequency of Physical Activity										
Age	Depression Life	Total	Never	Occasional	Several/month	Several/week	*x* ^2^	*df*	p*	*V*
Young people	Yes	93 (3.2)	46 (5.3) a	27 (3.0) b	6 (1.3) b	14 (2.0) b	20.81	3	<0.001	0.085
Young adults	Yes	334 (5.9)	159 (8.5) a	104 (5.3) b	31 (4.1) b	40 (3.9) b	35.77	3	<0.001	0.080
Older Adults	Yes	622 (10.7)	281 (13.8) a	241 (9.6) b	39 (7.3) b	61 (8.1) b	36.30	3	<0.001	0.079
Older	Yes	869 (14.8)	472 (19.8) a	312 (12.1) b	35 (9.2) b	50 (9.5) b	82.69	3	<0.001	0.119
Total	Yes	1918 (9.5)	958 (13.4) a	684 (8.6) b	111 (5.2) c	165 (5.5) c	235.2	3	<0.001	0.108
Age	Depression 12 months	Total	Never	Occasional	Several/month	Several/week	*x* ^2^	*df*	p*	*V*
Young people	Yes	65 (2.2)	30 (3.4) a	20 (2.2) ab	4 (0.9) b	11 (1.6) b	11.14	3	0.011	0.062
Young adults	Yes	237 (4.2)	118 (6.3) a	79 (4.0) b	17 (2.3) c	23 (2.2) c	37.69	3	<0.001	0.082
Older Adults	Yes	464 (8.0)	221 (10.9) a	180 (7.2) b	25 (4.7) c	38 (5.0) c	42.68	3	<0.001	0.086
Older	Yes	641 (10.9)	375 (15.7) a	215 (8.3) b	18 (4.7) c	33 (6.3) b.c	100.5	3	<0.001	0.131
Total	Yes	1407 (6.9)	744 (10.4) a	494 (6.2) b	64 (3.0) c	105 (3.5) c	244.6	3	<0.001	0.110
Age	Diagnosed Depression	Total	Never	Occasional	Several/month	Several/week	*x* ^2^	*df*	p*	*V*
Young people	Yes	81 (2.8)	40 (4.6) a	22 (2.5) b	5 (1.1) b	14 (2.0) b	17.30	3	0.001	0.077
Young adults	Yes	305 (5.4)	150 (8.0) a	90 (4.6) b	27 (3.6) b	38 (3.7) b	38.35	3	<0.001	0.082
Older Adults	Yes	573 (9.8)	263 (13.0) a	221 (8.8) b	36 (6.7) b	53 (7.0) b	38.00	3	<0.001	0.081
Older	Yes	788 (13.4)	432 (18.1) a	278 (10.8) b	32 (8.4) b	46 (8.8) b	78.56	3	<0.001	0.116
Total	Yes	1747 (8.6)	885 (12.4) a	611 (7.7) b	100 (4.7) c	151 (5.0) c	226.93	3	<0.001	0.106
Age	Antidepressants	Total	Never	Occasional	Several/month	Several/week	*x* ^2^	*df*	p*	*V*
Young people	Yes	47 (1.6)	23 (2.6) a	11 (1.2) b	5 (1.1) ab	8 (1.2) b	8.31	3	0.040	0.053
Young adults	Yes	197 (3.5)	100 (5.3) a	63 (3.2) b	15 (2.0) b.c	19 (1.8) c	32.79	3	<0.001	0.076
Older Adults	Yes	386 (6.6)	192 (9.5) a	151 (6.0) b	19 (3.5) c	24 (3.2) c	51.01	3	<0.001	0.094
Older	Yes	485 (8.2)	276 (11.5) a	172 (6.7) b	15 (3.9) c	22 (4.2) c	63.73	3	<0.001	0.104
Total	Yes	1115 (5.5)	591 (8.2) a	397 (5.0) b	54 (2.5) c	73 (2.4) c	199	3	<0.001	0.099

The data are presented by absolute and relative frequencies; Depression (Depression diagnosed by a physician); Non-depression (Depression no diagnosed); Antidepressants (have taken antidepressants in the last two weeks); No antidepressants (have not taken antidepressants in the last two weeks); Frequency of PA (“Which of these possibilities best describes the frequency with which you do some physical activity in your free time?”); *n* (participants). p* (*p*-value. Chi-square statistic); *df* (degree freedom); *V* (Cramer’s V); abc (Each subscript corresponds to significant differences between column proportions at 95%).

**Table 7:** Relationship between the prevalence of depression (lifetime, past 12 years and diagnosed) and days of physical activity in the general Spanish population of the EESE 2020 between 18 and 84 years, by age group.

Days of Physical Activity per Week										
Age	Depression Life	Total	0 days/week	1–2 days/week	3–4 days/week	5+ days/week	*x* ^2^	*df*	p*	*V*
Young people	Yes	93 (3.2)	60 (5.3) a	11 (2.4) b	13 (1.7) b	9 (1.7) b	20.81	3	<0.001	0.085
Young adults	Yes	334 (6.0)	205 (8.0) a	50 (5.4) b	40 (3.3) c	39 (4.2) b.c	35.77	3	<0.001	0.080
Older Adults	Yes	618 (10.7)	384 (12.7) a	60 (7.8) b	75 (8.3) b	99 (9.0) b	36.30	3	<0.001	0.079
Older	Yes	867 (14.8)	633 (17.2) a	60 (16.5) a	60 (10.9) b	114 (9.2) b	82.69	3	<0.001	0.119
Total	Yes	1912 (9.5)	1282 (12.3) a	181 (7.2) b	188 (5.5) c	261 (6.8) b	235.2	3	<0.001	0.108
Age	Depression 12 months	Total	0 days/week	1–2 days/week	3–4 days/week	5+ days/week	*x* ^2^	*df*	p*	*V*
Young people	Yes	65 (2.2)	40 (3.5) a	9 (2.0) a.b	7 (0.9) b	9 (1.7) b	26.01	3	<0.001	0.095
Young adults	Yes	237 (4.2)	156 (6.1) a	30 (3.2) b	27 (2.3) b	24 (2.6) b	39.70	3	<0.001	0.084
Older Adults	Yes	461 (7.9)	295 (9.8) a	37 (4.8) b	52 (5.7) b	77 (7.0) b	29.13	3	<0.001	0.071
Older	Yes	639 (10.9)	494 (13.4) a	43 (11.8)	40 (7.3) b	62 (5.0) b	54.18	3	<0.001	0.096
Total	Yes	1402 (7.0)	985 (9.5) a	119 (4.7) b	126 (3.7) c	172 (4.5) b.c	206.90	3	<0.001	0.101
Age	Diagnosed Depression	Total	0 days/week	1–2 days/week	3–4 days/week	5+ days/week	*x* ^2^	*df*	p*	*V*
Young people	Yes	81 (2.8)	51 (4.5) a	11 (2.4) a.b	10 (1.3) b	9 (1.7) b	22.73	3	0.001	0.085
Young adults	Yes	305 (5.4)	187 (7.3) a	44 (4.8) b	37 (3.1) c	37 (4.0) b.c	35.03	3	<0.001	0.079
Older Adults	Yes	569 (9.8)	359 (11.9) a	53 (6.9) b	70 (7.7) b	87 (7.9) b	31.63	3	<0.001	0.074
Older	Yes	786 (13.5)	577 (15.6) a	57 (15.7) a	54 (9.8) b	98 (7.9) b	55.55	3	<0.001	0.098
Total	Yes	1741 (8.6)	1174 (11.3) a	165 (6.6) b	171 (5.0) c	231 (6.1) b	195.44	3	<0.001	0.099
Age	Antidepressants	Total	0 days/week	1–2 days/week	3–4 days/week	5+ days/week	*x* ^2^	*df*	p*	*V*
Young people	Yes	47 (1.6)	28 (2.5) a	11 (2.4) a	4 (0.5) b	4 (0.7) b	15.25	3	0.002	0.073
Young adults	Yes	197 (3.5)	126 (4.9) a	25 (2.7) b	23 (1.9) b	23 (2.5) b	28.84	3	<0.001	0.072
Older Adults	Yes	384 (6.6)	254 (8.4) a	31 (4.0) b	44 (4.8) b	55 (5.0) b	33.91	3	<0.001	0.076
Older	Yes	483 (8.3)	367 (9.9) a	37 (10.2) a	31 (5.6) b	48 (3.9) b	51.99	3	<0.001	0.094
Total	Yes	1115 (5.5)	775 (7.4) a	104 (4.1) b	102 (3.0) c	130 (3.4) b.c	158.6	3	<0.001	0.089

The data are presented by absolute and relative frequencies; Depression (Depression diagnosed by a physician); Non-depression (Depression no diagnosed); Antidepressants (have taken antidepressants in the last 2 weeks); No antidepressants (have not taken antidepressants in the last two weeks); Days PA (how many days do you practice sport, gymnastics, cycling, brisk walking, etc., at least 10 min at a time); p* (*p*-value. Chi-square statistic); *df* (degree freedom); *V* (Cramer’s V); abc (Each subscript corresponds to significant differences between column proportions at 95%); *n* (participants).

**Table 8:** Relationship between the prevalence of depression (lifetime, past 12 years and diagnosed) and days of muscle-strengthening training per week in the general Spanish population of EESE 2020 between 18 and 84 years, by age group.

Muscle Strengthening Days per Week										
Age	Depression Life	Total	0 days/week	1–2 days/week	3–4 days/week	5+ days/week	*x* ^2^	*df*	p*	*V*
Young people	Yes	93 (3.2)	79 (4.2) a	3 (0.9) b	6 (1.4) b	5 (2.4) a.b	16.73	3	0.001	0.076
Young adults	Yes	333 (6.0)	283 (6.7) a	21 (3.6) b	23 (4.4) a.b	6 (2.5) b	16.83	3	0.001	0.055
Older Adults	Yes	618 (10.7)	558 (11.3) a	23 (6.5) b	26 (9.1) a.b	11 (6.0) b	13.25	3	0.004	0.048
Older	Yes	867 (14.9)	817 (15.3) a	14 (8.0) b	17 (10.6) a.b	19 (12.2) a.b	10.67	3	0.014	0.043
Total	Yes	1911 (9.5)	1737 (10.6) a	61 (4.2) b	72 (5.2) b	41 (5.2) b	117.6	3	<0.001	0.077
Age	Depression 12 months	Total	0 days/week	1–2 days/week	3–4 days/week	5+ days/week	*x* ^2^	*df*	p*	*V*
Young people	Yes	65 (2.3)	54 (2.8) a	1 (0.3) b	5 (1.2) b.c	5 (2.5) a. c	11.49	3	0.009	0.063
Young adults	Yes	237 (4.2)	204 (4.8) a	11 (1.9) b	17 (3.3) a.b	5 (2.1) b	15.19	3	0.002	0.052
Older Adults	Yes	461 (8.0)	419 (8.4) a	14 (3.9) b	20 (7.0) a.b	8 (4.4) a.b	12.98	3	0.005	0.047
Older	Yes	639 (11.0)	612 (11.5) a	9 (5.1) b	10 (6.3) b	8 (5.2) b	16.60	3	0.001	0.053
Total	Yes	1402 (7.0)	1289 (7.8) a	35 (2.4) b	52 (3.7) c	26 (3.3) b.c	105.4	3	<0.001	0.072
Age	Diagnosed Depression	Total	0 days/week	1–2 days/week	3–4 days/week	5+ days/week	*x* ^2^	*df*	p*	*V*
Young people	Yes	81 (2.8)	69 (3.6) a	1 (0.3) b	6 (1.4) b	5 (2.4) a.b	16.21	3	0.001	0.075
Young adults	Yes	304 (5.4)	259 (6.1) a	17 (2.9) b	22 (4.2) a.b	6 (2.5) b	16.28	3	0.001	0.054
Older Adults	Yes	569 (9.8)	514 (10.4) a	20 (5.6) b	26 (9.1) a.b	9 (4.9) b	13.86	3	0.003	0.049
Older	Yes	786 (13.5)	742 (13.9) a	13 (7.4) b	16 (10.0) a.b	15 (9.6) a.b	10.14	3	0.017	0.042
Total	Yes	1740 (8.7)	1584 (9.6) a	51 (3.5) b	70 (5.0) c	35 (4.4) b.c	110.64	3	<0.001	0.074
Age	Antidepressants	Total	0 days/week	1–2 days/week	3–4 days/week	5+ days/week	*x* ^2^	*df*	p*	*V*
Young people	Yes	47 (1.6)	36 (1.9) a	2 (0.6) a	4 (0.9) a	5 (2.4) a	5.29	3	0.152	0.043
Young adults	Yes	196 (3.5)	167 (3.9) a	13 (2.2) b	10 (1.9) b	6 (2.5) a.b	9.61	3	0.022	0.041
Older Adults	Yes	384 (6.6)	350 (7.1) a	12 (3.4) b	14 (4.9) a.b	8 (4.4) a.b	10.45	3	0.015	0.043
Older	Yes	483 (8.3)	458 (8.6) a	11 (6.2) a.b	6 (3.7) b	8 (5.1) a.b	8.07	3	0.045	0.037
Total	Yes	1110 (5.5)	1011 (6.1) a	38 (2.6) b	34 (2.4) b	27 (3.4) b	68.59	3	<0.001	0.058

The data are presented by absolute and relative frequencies; Depression (Depression diagnosed by a physician); Non-depression (Depression no diagnosed); Antidepressants (have taken antidepressants in the last 2 weeks); No antidepressants (have not taken antidepressants in the last two weeks); Strengthening days (how many days do you do activities specifically aimed at strengthening your muscles); p* (*p*-value. Chi-square statistic); *df* (degree freedom); *V* (Cramer’s V); abc (Each subscript corresponds to significant differences between column proportions at 95%); *n* (participants).

## Data Availability

The data used were obtained from public use files, which are available on the website of the Spanish Ministry of Health, Consumer Affairs, and Social Welfare: https://www.sanidad.gob.es/estadEstudios/estadisticas/EncuestaEuropea/Enc_Eur_Salud_en_Esp_2020.htm (accessed on 5 November 2021).
